# Complete Genome Sequences of Three *Mycoplasma anserisalpingitis* (*Mycoplasma* sp. 1220) Strains

**DOI:** 10.1128/MRA.00985-19

**Published:** 2019-09-12

**Authors:** Dénes Grózner, Barbara Forró, Áron Botond Kovács, Szilvia Marton, Krisztián Bányai, Zsuzsa Kreizinger, Kinga Mária Sulyok, Miklós Gyuranecz

**Affiliations:** a Institute for Veterinary Medical Research, Centre for Agricultural Research, Hungarian Academy of Sciences, Budapest, Hungary; b Department of Microbiology and Infectious Diseases, University of Veterinary Medicine, Budapest, Hungary; Broad Institute

## Abstract

Mycoplasma anserisalpingitis is a goose pathogen. The main symptoms in affected flocks are inflammation of the cloaca and the reproductive organs, decreased egg production, and increased embryo mortality. Here, we report the complete genome sequences of the type strain (ATCC BAA-2147) and two clinical isolates.

## ANNOUNCEMENT

Mycoplasma anserisalpingitis (also known as Mycoplasma sp. 1220) is commonly found in geese (1). This species frequently cooccurs with Mycoplasma anseris and Mycoplasma cloacale, leading to diseases in ganders and layers (1, 2). Cloaca and phallus inflammation, salpingitis, decreased egg production, and lethal pathological changes in the embryos are the main symptoms (3). Hence, airsacculitis and peritonitis are also common, and several general symptoms in affected flocks have also been described (3, 4). The type strain was isolated from a goose flock in Hungary with a history of phallus and cloaca inflammation (5). Two *M*. *anserisalpingitis* strains—designated MYCAV 93 and MYCAV 177—were isolated from the inflamed phalluses of ganders, also in Hungary.

The M. anserisalpingitis type strain (ATCC BAA-2147) was purchased directly from the repository. We obtained the MYCAV 93 and MYCAV 177 field isolates during routine diagnostic examinations of domestic geese in 2011 and 2015, respectively. Isolation and cell propagation were performed in Oxoid *Mycoplasma* broth medium (pH 7.8) (Thermo Fisher Scientific, Inc., Waltham, MA) supplemented with 0.5% (wt/vol) sodium pyruvate, 0.5% (wt/vol) glucose, and 0.005% (wt/vol) phenol red and were incubated at 37°C. DNA was extracted with the QIAamp DNA minikit (Qiagen, Inc., Hilden, Germany).

DNA library preparations and genome sequencing results are summarized in Table 1. NxTrim 0.4.3 software (6) with default settings was used to trim the junction adapters from all of the raw mate pair (MP) reads, generating shorter paired-end (PE) reads as well. First, contigs were generated for each strain from all of the paired-end output data using the SPAdes Genome Assembler 3.11 (7) with the “assembly only” option. Then, the paired-end contigs and the trimmed mate pair output data were assembled with the same option, generating the draft genomes. Trimmed reads (mate pair and paired end) were control mapped to the draft *de novo* genome and curated with Geneious 9.1.8 software (8). Circularization of the contigs was performed by primer pairs and PCR assays specific for the contig ends (https://doi.org/10.6084/m9.figshare.9724238), and the PCR products were sequenced on an ABI Prism 3100 automated DNA sequencer (Applied Biosystems, Foster City, CA). The NCBI Prokaryotic Genome Annotation Pipeline (9) online service was used to annotate the genomes.

**TABLE 1:** Sequencing methods, genome information, and database accession numbers of *Mycoplasma anserisalpingitis* strains ATCC BAA-2147, MYCAV 93, and MYCAV 177

*M. anserisalpingitis* strain	GenBank accession no.	SRA no.	Total coverage (×)	Size (bp)	G+C content (%)	No. of CDS^*d*^	No. of rRNAs	No. of tRNAs	DNA library prepn kit^*a*^	Illumina equipment^*a*^	Generated reads^*b*^	SRA accession no.	No. of trimmed reads	Coverage (×)
ATCC BAA-2147	CP042295	PRJNA554588	578	959,110	26.6	774	6	32	TruSeq LT	MiSeq	2 × 250-bp MP	SRX6441455	3,110,956^*c*^	NA^*e*^
MYCAV 93	CP041663	PRJNA553666	753	919,993	26.7	730	4	32	Nextera mate pair	NextSeq 500	2 × 150-bp MP	SRX6426231	1,829,740^*c*^	216
									Nextera XT	NextSeq 500	2 × 75-bp PE	SRX6426230	7,092,604	534
MYCAV 177	CP041664	PRJNA554567	1,288	908,787	26.7	742	6	32	TruSeq LT	MiSeq	2 × 250-bp MP	SRX6440844	7,507,396^*c*^	1,520
									Nextera XT	NextSeq 500	2 × 75-bp PE	SRX6440843	6,971,896	530

a Illumina, Inc. (San Diego, CA, USA).

b MP, mate pair reads; PE, paired-end reads.

c Mate pair and generated paired-end reads.

d CDS, coding sequences.

e NA, not applicable.

For detailed results concerning the annotated genomes, see Table 1. Whole-genome alignment with Mauve 2.3.1 software (10) integrated into Geneious showed that the three strains have similar genomic structures, except in one case. A 125-kb inversion compared to the others was revealed in the genome of MYCAV 93 (positions 177,243 to 302,855), but genomic rearrangement was confirmed within other *Mycoplasma* species as well (11, 12).

We hope that the complete genomes presented here will improve research on this commercially important waterfowl *Mycoplasma* species.

### Data availability.

The annotated genome sequences were deposited in GenBank, and the raw read data are available in the Sequence Read Archive. The accession numbers are listed in Table 1.
